# Activation of the KDM5A/miRNA-495/YTHDF2/m6A-MOB3B axis facilitates prostate cancer progression

**DOI:** 10.1186/s13046-020-01735-3

**Published:** 2020-10-21

**Authors:** Chen Du, Caihong Lv, Yue Feng, Siwen Yu

**Affiliations:** 1 Department of Urology Surgery, Harbin Medical University Cancer Hospital, No. 150, Haping Road, Nangang District, Harbin, 150000 Heilongjiang Province People’s Republic of China; 2 Department of Laboratory, the Fourth Affiliated Hospital of Harbin Medical University, Harbin, 150001 People’s Republic of China

**Keywords:** KDM5A, microRNA-145, YTHDF2, MOB3B, m6A modification, Prostate cancer, Migration, Invasion

## Abstract

**Background:**

Accumulating evidence supports that lysine-specific demethylase 5 (KDM5) family members act as oncogenic drivers. This study was performed to elucidate the potential effects of KDM5A on prostate cancer (PCa) progression via the miR-495/YTHDF2/m6A-MOB3B axis.

**Methods:**

The expression of KDM5A, miR-495, YTHDF2 and MOB3B was validated in human PCa tissues and cell lines. Ectopic expression and knockdown experiments were developed in PCa cells to evaluate their effects on PCa cell proliferation, migration, invasion and apoptosis. Mechanistic insights into the interaction among KDM5A, miR-495, YTHDF2 and MOB3B were obtained after dual luciferase reporter, ChIP, and PAR-CLIP assays. Me-RIP assay was used to determine m6A modification level of MOB3B mRNA in PCa cells. Mouse xenograft models of PCa cells were also established to monitor the tumor growth.

**Results:**

KDM5A was highly expressed in human PCa tissues and cell lines. Upregulated KDM5A stimulated PCa cell proliferation, migration and invasion, but reduced cell apoptosis. Mechanistically, KDM5A, as a H3K4me3 demethylase, bound to the miR-495 promoter, which led to inhibition of its transcription and expression. As a target of miR-495, YTHDF2 could inhibit MOB3B expression by recognizing m6A modification of MOB3B mRNA and inducing mRNA degradation. Furthermore, KDM5A was found to downregulate MOB3B expression, consequently augmenting PCa cell proliferation, migration and invasion in vitro and promoting tumor growth in vivo via the miR-495/YTHDF2 axis.

**Conclusion:**

In summary, our study highlights the potential of histone demethylase KDM5A activity in enhancing PCa progression, and suggests KDM5A as a promising target for PCa treatment.

**Supplementary information:**

**Supplementary information** accompanies this paper at 10.1186/s13046-020-01735-3.

## Background

Prostate cancer (PCa) is the most common malignancy in males and a major cause of mortality worldwide, causing approximately 1.6 million incident cases and 366,000 deaths each year [1]. Risk factors for this disease include advancing age, race, genetics, obesity, physical activity, smoking and occupation [2]. There are multiple management options for men with PCa, such as surgery, radiation, chemotherapy, vaccines, hormonal therapeutics, and bone-targeting agents [3]. Despite the efficacy these approaches demonstrated, novel means to assess the presence of PCa, monitor its progression and predict its outcome at an early stage in a reliable manner are still required, which necessitate a better understanding of its underlying molecular processes.

Lysine-specific demethylase 5A (KDM5A), known as a histone H3K4 demethylase [4], has recently become a promising therapeutic target for cancers due to its key roles in important cancer processes including tumorigenesis, metastasis, and drug tolerance [5]. Cui et al. reported that KDM5A could stimulate pancreatic cancer cell proliferation in vitro and tumor growth in vivo by suppressing the expression of mitochondrial pyruvate carrier 1 (MPC-1) [6]. KDM5A was also found to be significantly associated with tumor stage progress and metastasis in patients with clear cell renal cell carcinoma [7]. Upregulated KDM5A has been demonstrated in prostate tissues, but its downstream mechanisms remain enigmatic [8].

In addition, abnormal expression of microRNAs (miRs or miRNAs) is also implicated in PCa progression. miR-495 has been identified as a tumor suppressor miRNA in PCa owing to its inhibitory effect on Akt and mTOR, thus it further suppressing cancer cell proliferation, migration, and invasion in vitro [9]. The silico analysis in the present study revealed that miR-495 could directly bind to the mRNA of YTH domain family 2 (YTHDF2), a member of the YTH domain family and the first discovered m6A reader protein, knockdown of which significantly reduces cell proliferation and migration of PCa DU-145 and PC3 cell lines [10]. Moreover, YTHDF2 showed a regulatory role in mouse neural development by promoting m6A-dependent degradation of neural development-related mRNA targets, including mps one binder kinase activator 3B (MOB3B) [11]. MOB3B is a member of the MOBs family that is highly conserved in the eukaryotic species and can act as signal transducers in essential intracellular pathways and have diverse cancer-associated cellular functions [12]. Hence, based on the aforementioned information, we hypothesized that KDM5A could participate in the development of PCa via the miR-495/YTHDF2/m6A-MOB3B signaling axis. We therefore performed in vitro and in vivo experiments to verify the clinical signification of the KDM5A/miR-495/YTHDF2/m6A-MOB3B signaling in the development of PCa.

## Materials and methods

### Ethics statement

The current study was approved by the Ethics Committee of Harbin Medical University Cancer Hospital and performed in strict accordance with the *Declaration of Helsinki*. All participants signed informed consent prior to enrollment. Extensive efforts were made to ensure minimal usage of animals as well as their suffering.

### Study subjects

A total of 78 patients who received radical prostatectomy and transurethral resection of the prostate at the Department of Urology of Harbin Medical University Cancer Hospital from June 2014 to June 2016 were recruited in our study. None of these patients received anti-tumor treatment before surgery. The cancer tissue samples without necrosis or hemorrhage were biopsied during surgery with adjacent normal tissues (no cancer cells confirmed by pathological examination) and stored in a − 80 °C freezer. The demographic information of patients was extracted and collected from the medical record system, and all patients were followed up post-surgery in order to understand the clinical outcomes after treatment and obtain comprehensive clinical data of patients. The follow-up ended in December 2019, with a total of 3–36 months. Kaplan-Meier method was used to analyze the correlation between KDM5A expression and total survival (OS) and progression-free survival (DFS).

### Immunohistochemistry

Paraffin sections of tumor tissues from each group were taken for immunohistochemical analysis, which were dewaxed, dehydrated by alcohol gradient, and immersed in 3% methanol H_2_O_2_ for 20 min. Antigen was repaired through water-bath of repair solution. Sections were then blocked with normal goat serum blocking solution (C-0005, Haoran Bio, Shanghai, China) and placed at room temperature for 20 min to dry the slides. Next, sections were incubated with primary antibody to KDM5A (ab92533, 1:500) overnight at 4 °C. The secondary antibody goat anti-rabbit immunoglobulin G (IgG) was then added into the sections and placed at 37 °C for 20 min. After addition of horseradish peroxidase labeled Streptomyces ovalbumin working solution (0343-10,000 U, ImunBio, Beijing, China) the sections were placed at 37 °C for 20 min. Color was developed with diaminobenzidine (DAB; ST033, Whiga Biotechnology, Guangzhou, China) and sections were counterstained with hematoxylin (PT001, Bogoo, Shanghai) for 1 min, turned blue with 1% ammonia water and dehydrated by gradient alcohol. After regular permeabilization and mounting, sections were observed under a microscope with each section randomly selected under five high power field of view. A total of 100 cells were counted in each field. Positive cells < 10% was taken for negative, while positive cells ≥10% and < 50% for positive, and positive cells > 50% was considered as strong positive.

### RNA isolation and quantitation

The total RNA was extracted from cancer tissues and cells using the TRIzol reagent (Invitrogen, Carlsbad, CA, USA). The RNA was then reversely transcribed into complementary DNA using the TaqMan MicroRNA Assays Reverse Transcription primer (4,427,975, Applied Biosystems, USA)/PrimeScript RT reagent Kit (RR047A, Takara, Japan). The primers for KDM5A, miR-495, YTHDF2 and MOB3B were designed and synthesized by Takara Holdings Inc. (Kyoto, Japan) (Table 1). Subsequently, reverse transcription quantitative polymerase chain reaction (RT-qPCR) was conducted on an ABI 7500 instrument (Applied Biosystems, Foster City, CA, USA). The fold changes were calculated using the 2^-ΔΔCt^ method with U6 and glyceraldehyde-3-phosphate dehydrogenase (GAPDH) serving as internal references, respectively.

**Table 1:** Primer sequences for RT-qPCR

Gene	Sequence
KDM5A	Forward: 5′-TTACCAACAGGTCAGACGCAT-3′
	Reverse: 5′-GGTTTGCTACATTCCTCGGCG-3′
miR-495	Forward: 5′-GGGGAAACAAACATGGTGCAC-3′
	Reverse: 5′-CAGTGCGTGTCGTGGAGT-3′
YTHDF2	Forward: 5′-CATGAATGGGAAGGGTCCCG-3′
	Reverse: 5′-GACGAATGTGTCGCAGTTGG-3′
MOB3B	Forward: 5′-GTGGCAGGATGATCTCAA-3′
	Reverse: 5′-CGGCACAGGATCTTCTTG-3′
U6	Forward: 5′-CTCGCTTCGGCAGCACA-3′
	Reverse: 5′-AACGCTTCACGAATTTGCGT-3′
GAPDH	Forward: 5′-CTGGGCTACACTGAGCACC-3′
	Reverse: 5′-AAGTGGTCGTTGAGGGCAATG-3′

### Western blot analysis

Total protein was extracted from cancer cells and tissues using radio-immunoprecipitation assay lysis buffer, with the protein concentration then determined with a bicinchoninic acid kit. The protein was separated and transferred onto a polyvinylidene fluoride membrane. The membrane was then blocked and underwent overnight incubation with primary antibodies against KDM5A (1:10000), H3K4me3 (1 μg/mL, rabbit, Abcam, USA), YTHDF2 (1:2000, rabbit) and MOB3B (1:1000, rabbit, Sigma-Aldrich Chemical Company, St Louis, MO, USA). The next day, the membrane was re-probed with horseradish peroxidase-labeled secondary goat anti-rabbit IgG (1:1000, Santa Cruz Biotechnology, Inc., Santa Cruz, CA, USA). Afterwards, the membrane was visualized and the protein bands were quantified using the Bio-Rad ChemiDoc™ system and ImageJ2x software. The ratio of the gray value of the target band to GAPDH (1:10000, rabbit, Santa Cruz Biotechnology, Inc., Santa Cruz, CA, USA) was representative of the relative protein expression.

### Cell culture and transfection

PCa cell lines (LNCaP, C42, DU145 and PC3) were purchased from American Type Culture Collection (ATCC; Manassas, VA, USA). The normal human prostate epithelial cell line RWPE-1 (Oulu Biotechnology, Shanghai, China) served as a control, and were cultured in Roswell Park Memorial Institute (RPMI) 1640 medium (Gibco, USA) containing 10% fetal bovine serum (Gibco, USA), 100 μg/mL streptomycin and 100 U/mL penicillin in a 5% CO_2_ incubator (Thermo Fisher Scientific Inc., Waltham, MA, USA) at 37 °C. Upon reaching approximately 75% confluence, the cells were transiently transfected using Lipofectamine 2000 (Invitrogen) with the following plasmids purchased from Sino Biological, Inc. (Beijing, China): overexpression (oe)-KDM5A, short hairpin RNA (sh)-KDM5A (sh1-KDM5A [5′-GGAACUGGGUCUCUUUUGA-3′], sh2-KDM5A [5′-GCAAAUGAGACAACGGAAA-3′] and sh3-KDM5A [5′-UGACAAUGGUGGACCGCAU-3′]), oe-KDM5A + miR-495 mimic, miR-495 mimic, miR-495 inhibitor, miR-495 mimic + oe-YTHDF2 and oe-KDM5A + oe-MOB3B as well as their corresponding controls (oe-negative control [NC], sh-NC, oe-NC + mimic NC, oe-KDM5A + mimic NC, mimic NC, inhibitor NC, mimic NC + oe-NC, miR-495 mimic + oe-NC, mimic NC + oe-YTHDF2 and oe-KDM5A + oe-NC). After 6 h of transfection, the cells were cultured for 48 h and then collected.

### Colony formation assay for cell proliferation assay

PCa cells were inoculated into dishes containing 10 mL preheated culture medium, and evenly dispersed by gentle rotation, followed by culture, with the culture medium changed once every 2–3 days. The culture was halted once the clone in the dish was visible to the naked eyes. After removal of supernatant, the cells were washed, and fixed. The fixed cells were stained with an appropriate amount of GIMSA (Invitrogen, USA) for 10–30 min. The number of cell clones was counted under an inverted microscope (Leica DMi8-M, Co. Ltd., Solms, Germany), and the colony formation rate was calculated using the formula: the number of cell clones/the number of inoculated cells × 100%.

### Flow cytometry

Annexin V-fluorescein isothiocyanate (FITC)/propidium iodide (PI) double staining method was used to detect cell apoptosis. The PCa cells were inoculated into 6-well plates at a density of 2 × 10^5^ cells/well. After cell transfection, the culture medium was removed, and the cells were trypsinized and collected in a centrifuge tube for centrifugation at 800 g, with the supernatant discarded. According to the instructions of the Annexin V-FITC Apoptosis Detection Kit (BD Biosciences, San Jose, CA, USA), the cells were resuspended, and added with 5 μL FITC and 5 μL PI under dark conditions. At last, the cell apoptosis was detected using BD FACSCalibur.

### Transwell assay

PCa cells were starved, then digested, and resuspended in serum-free Opti-MEMI medium (Invitrogen, USA). The cell suspension was then seeded into the 8-μm Transwell chamber (Corning, NY, USA) in a 24-well plate (100 μL per chamber, a total of 3 chambers). Next, 600 μL 10% RPMI-1640 medium was added to the lower chamber and incubated. For the cell migration experiment, the cells were fixed, after which the chamber was treated with 0.2% Triton X-100 solution (Sigma-Aldrich Chemical Company, St Louis, MO, USA), and stained with crystal violet.

During cell invasion experiment, 50 μL Matrigel (Sigma-Aldrich Chemical Company, St Louis, MO, USA) settled in the chamber before the experiment. After 48 h, fixation and staining were conducted with the aforementioned procedures. The number of stained cells was counted in five randomly selected visual fields under an inverted microscope (Leica DMi8-M, Co. Ltd., Solms, Germany) to obtain the mean value.

### Scratch test

After 48 h of transfection, PCa cells were seeded into 6-well plates at a density of 5 × 10^5^ cells/well. After the cells completely adhered to the bottom, a scratch was made in the middle of each well using a 2-mm cell scraper and the cell culture was continued for 24 h. The cells were photographed at 0 h and 24 h after scratch, and the rate of scratch healing was calculated with the Image-Pro Plus 6.0 software (Media Cybernetics, Inc., Silver Spring, MD, USA).

### Dual luciferase reporter assay

The predicted binding site and mutation fragments of miR-495 promoter with KDM5A were inserted into luciferase reporter vectors (Beijing Huayueyang Biotechnology Co., Ltd., Beijing, China), known as reporter plasmids miR-495 promoter-wild-type (WT) (with sequence of 3′-CAGTGACCCA-5′) and miR-495 promoter-mutant (MUT) (with sequence of 3′-TGAGAGTATG-5′). Oe-NC and oe-KDM5A were co-transfected into 293 T cells (Oulu Biotechnology, China) with miR-495-WT and miR-495-MUT plasmids in order to detect whether KDM5A could bind with KDM5A. After 48 h of transfection, cells were collected and lysed. The luciferase activity was detected with the use of a luciferase assay kit (K801–200, Biovision, Bay Area, San Francisco, USA) on the analysis system (Promega Corporation, Madison, WI, USA). With renilla luciferase as a loading control, the luciferase activity was calculated as the relative luciferase unit (RLU) activity of firefly luciferase/RLU activity of renilla luciferase. Likewise, the interaction between miR-495 and YTHDF2 was detected in the same way.

### Chromatin immunoprecipitation (ChIP) assay

After 48 h of transfection, PCa cells from each group were fixed in 1% formaldehyde at 37 °C for 10 min, which was then terminated by adding glycine solution for a 5 min reaction on ice. After rinsing with PBS, the cells were incubated and centrifuged to obtain cell precipitate. The cells were resuspended in 200 μL SDS lysis buffer for 10 min on ice. The chromatin DNA was broken by ultrasound on ice. The cells were centrifuged at 14000 rpm for 10 min, after which the supernatant was diluted with ChIP buffer containing protease inhibitor, and with blocking solution added for a 30-min incubation at 4 °C. After centrifugation at 1000 rpm for 1 min, the supernatant was collected and divided into three parts, with one as Input, one being incubated with rabbit IgG antibody (Abcam, China) as the NC, and the other part incubated with rabbit KDM5A antibody (2 μg used for 25 μg of chromatin, Abcam, China) at 4 °C overnight. We added Protein G Dynabeads (Thermo Fisher Scientific Inc., Waltham, MA, USA) to the supernatant and incubated it at 4 °C for 1 h to precipitate antibody-transcription factor complex. After centrifugation at 1000 rpm for 1 min, the precipitate was washed and eluted with elution buffer. Twenty μL NaCl (5 mol/L) were added into the eluted precipitate and Input DNA respectively and bathed in water at 65 °C for 4 h to ravel the DNA cross-linking. DNA fragments were then purified and recovered post-incubation with protease K. Subsequently, RT-qPCR was conducted to detect the expression of miR-495 with the recovered DNA as a template.

### Methylated RNA binding protein immunoprecipitation (me-RIP) assay

Total RNA was extracted from tissues and cells using the TRIzol method. The mRNA in the total RNA was isolated and purified using the PolyATtract® mRNA Isolation Systems (A-Z5300, A&D Technology Corporation, Beijing, China). IP buffer supplemented with 20 mM Tris pH 7.5, 140 mM NaCl, 1% NP-40 and 2 mM EDTA was added m6A antibody (1:500, ab151230, Abcam, USA) or IgG antibody (ab109489, 1:100, Abcam, USA) and incubated with protein A/G magnetic beads for 1 h in order for binding. IP buffer with ribonuclease inhibitor and protease inhibitor was then added with the purified mRNA-bead-antibody complex and incubated overnight at 4 °C. The RNA was eluted with elution buffer, and purified with phenol-chloroform. MOB3B was analyzed by RT-qPCR.

### Photo-activatable ribonucleoside-enhanced crosslinking and immunoprecipitation (PAR-CLIP)

PCa tissues and cells were incubated with 200 mm 4-thiopyridine (Sigma, USA) for 14 h, and cross-linked with 0.4 J/cm^2^ at 365 nm. After cleavage, YTHDF2 antibody (5 mg and 3 mg respectively) was used for immunoprecipitation at 4 °C. The precipitated RNA was then labeled with [g-32-P]-ATP and observed by autoradiography. After protease K treatment, the RNA was extracted and MOB3B expression was detected by RT-qPCR.

### Xenograft tumor in nude mice

A total of 36 specific pathogen-free male Balb/c mice (aged 5 weeks; weighing 18–22 g) were purchased from Shanghai SLAC Laboratory Animal Co., Ltd. (Shanghai, China). PCa cells were resuspended in serum-free RPMI-1640 medium (Gibco, USA) to cell suspension at a density of 1 × 10^6^ cells/200 μL. The Balb/c nude mice were randomly divided into three groups (*n* = 12 in each group), and the tumorigenesis experiment lasted for 4 weeks. Under anesthesia with ether, the nude mice were disinfected and subcutaneously inoculated with cells transfected with oe-NC, oe-KDM5A + oe-NC and oe-KDM5A + oe-MOB3B at a density of 1 × 10^6^ cells/mouse (200 μL) at the back of the right hind leg. All nude mice were raised in the same environment, with themselves and their inoculation site being observed every day. The tumor size was measured every 7 days, and the length and width of the tumor were recorded, with the tumor volume estimated as length × width^2^/2. After 4 weeks, all mice were euthanized by cervical dislocation under anesthesia, and their tumor tissue was dissected, photographed and weighed.

### Statistical analysis

All data were processed using the SPSS 21.0 (IBM Corp. Armonk, NY, USA) with a level of *p* < 0.05 as statistical significance. Continuous data were demonstrated as mean ± standard deviation. Data with normal distribution and homogeneity of variance between two groups were compared using *t*-test. Comparison of the mean among multiple groups were analyzed by one-way analysis of variance (ANOVA) with Tukey’s post hoc test or repeated measures ANOVA with Bonferroni post hoc test. Kaplan-Meier method was used to calculate the survival rate and duration of the patients, with Log-rank test used for single factor analysis. Pearson correlation coefficient was utilized for correlation analysis.

## Results

### Upregulated KDM5A in PCa tissues and cells was linked to an unfavorable prognosis of patients with PCa

**Fig. 1:**
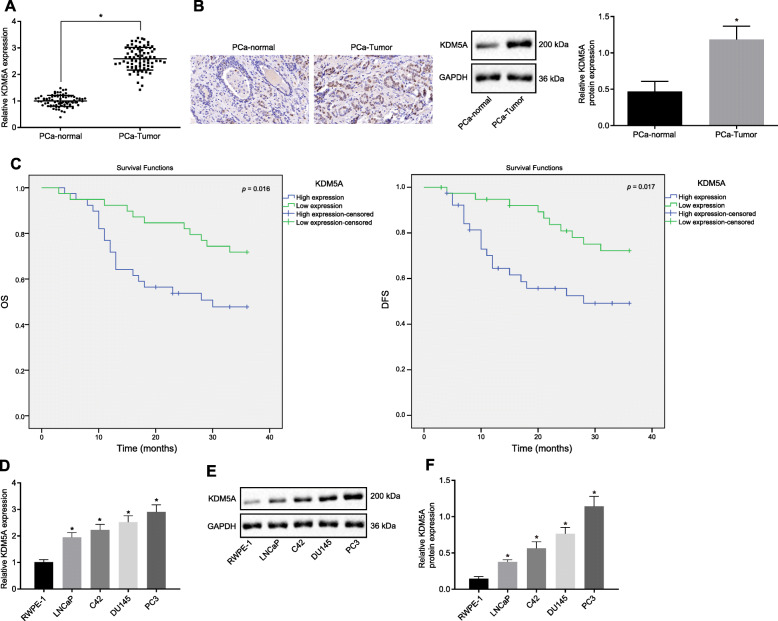
KDM5A is highly expressed in the PCa tissues and cells, and it predicts a poor prognosis. **a**, KDM5A mRNA expression in PCa and adjacent normal tissues of patients detected by RT-qPCR. *N* = 78 for PCa patients. **b**, Western blot analysis and immunohistochemistry of KDM5A protein in PCa and adjacent normal tissues of patients. *N* = 78 for PCa patients. **c**, Corelation of KDM5A expression with DFS and OS analyzed using the Kaplan-Meier method. **d**, KDM5A mRNA expression in RWPE-1, LNCaP, C42, DU145 and PC3 cells detected by RT-qPCR. **e** and **f**, Western blot analysis and quantification of KDM5A protein in RWPE-1, LNCaP, C42, DU145 and PC3 cells. * *p* < 0.05 vs. RWPE-1 cells. Data in panel A and B were analyzed by paired *t*-test and those in panel C-E were analyzed by one-way ANOVA with Tukey’s post hoc test

We first explored the possible role of KDM5A in the development of PCa. RT-qPCR, Western blots and immunohistochemistry revealed that the mRNA and protein expression levels of KDM5A were increased in cancer tissues compared to the adjacent normal tissues collected from the 78 PCa patients (*p* < 0.05; Fig. 1a, b). Next, KDM5A expression was grouped into high expression (> 2.593) and low expression (≤ 2.593), based on the mean expression value of KDM5A. Kaplan-Meier analysis showed lower OS and DFS of PCa patients with high KDM5A expression than the OS and DFS of patients with low KDM5A expression (Fig. 1c), suggesting that the high expression of KDM5A may correlate with poor prognosis of PCa patients. In addition, mRNA and protein levels of KDM5A were elevated in LNCaP, C42, DU145 and PC3 cells compared with RWPE-1 cells, with PC3 cells showing a more increased KDM5A expression (*p* < 0.05; Fig. 1d, e). Thus, PC3 cells were selected for the subsequent experiments. These results indicate that highly expressed KDM5A in PCa is related to a poor prognosis of patients with PCa.

### Overexpression of KDM5A promoted the proliferation, migration and invasion of PCa cells, while reducing cell apoptosis

**Fig. 2:**
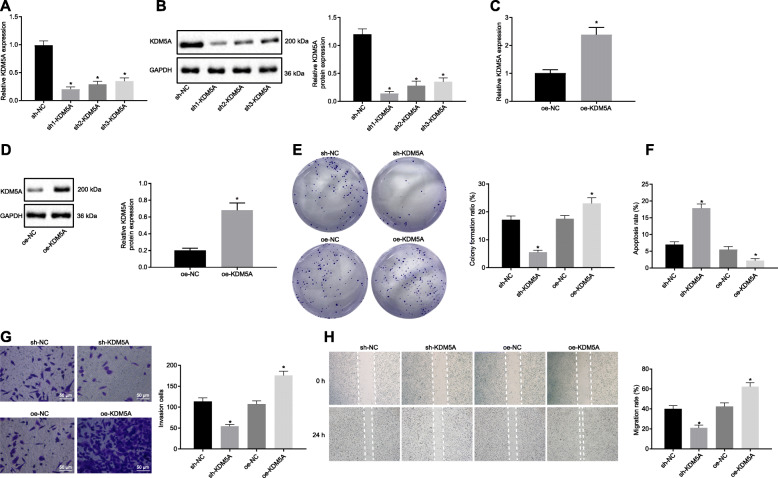
KDM5A stimulates PCa cell proliferation, migration and invasion while reducing cell apoptosis. **a** and **b**, The silencing efficiency of KDM5A in PC3 cells detected by RT-qPCR and Western blot analysis. **c**, KDM5A mRNA expression in oe-KDM5A-transfected cells detected by RT-qPCR. **d**, Western blots of KDM5A protein in oe-KDM5A-transfected cells. **e**, PC3 cell proliferation following oe- or sh-KDM5A transfection measured by colony formation assay. **f**, PC3 cell apoptosis following oe- or sh-KDM5A transfection measured by flow cytometry. **g**, PC3 cell invasion following oe- or sh-KDM5A transfection measured by Transwell assay. Scale bar = 50 μm. **h**, PC3 cell migration following oe- or sh-KDM5A transfection measured by Scratch test. * *p* < 0.05 vs. cells transfected with sh-NC or oe-NC. Data in panel A, B and E-H were analyzed by one-way ANOVA with Tukey’s post hoc test, and data in panel C and D were analyzed by unpaired *t*-test

We consequently became interested in the cell functions of PCa cells and the effect of KDM5A on the proliferation, migration, invasion and apoptosis of PC3 cells. The results of RT-qPCR and Western blots revealed a reduction in the mRNA and protein expression levels of KDM5A in cells transfected with sh1-KDM5A, sh2-KDM5A and sh3-KDM5A (*p* < 0.05), among which sh1-KDM5A showed a superior silencing efficiency (*p* < 0.05; Fig. 2a, b) and was therefore selected for subsequent experiments. Additionally, the mRNA and protein levels of KDM5A were significantly increased in cells following oe-KDM5A transfection (*p* < 0.05; Fig. 2c, d). Silencing of KDM5A was found to reduce PC3 cell proliferation, invasion and migration while inducing apoptosis (*p* < 0.05; Fig. 2e-h), which was undermined by overexpression of KDM5A. Collectively, upregulated KDM5A could augment the proliferation, migration and invasion of PCa cells, while diminishing the cell apoptosis.

### KDM5A repressed miR-495 expression by binding to its promoter region and subsequently stimulated PCa cell proliferation, migration and invasion

**Fig. 3:**
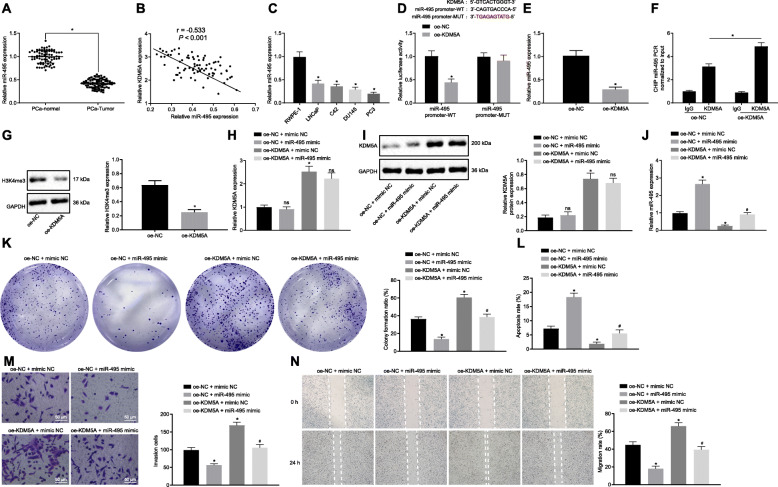
KDM5A potentiates PCa cell proliferation, invasion and migration while suppressing cell apoptosis by inhibiting miR-495. **a**, miR-495 expression in cancer and adjacent normal tissues of 78 patients detected by RT-qPCR. **b**, Correlation between miR-495 and KDM5A in cancer and adjacent normal tissues of 78 patients. **c**, miR-495 expression in RWPE-1, LNCaP, C42, DU145 and PC3 cells detected by RT-qPCR. **d**, The binding of KDM5A to the promoter of miR-495 confirmed by dual luciferase reporter assay. **e**, miR-495 expression in oe-KDM5A-transfected PC3 cells. **f**, The binding of KDM5A to the promoter of miR-495 verified by ChIP assay. **g**, Western blots of H3K4me3 in the antibody-transcription factor complex pulled down by KDM5A antibody. **h**, KDM5A mRNA expression in PC3 cells following varied transfection detected by RT-qPCR. **i**, Western blot analysis of KDM5A protein in PC3 cells following varied transfection. **j**, miR-495 expression in PC3 cells following varied transfection detected by RT-qPCR. **k**, PC3 cell proliferation following varied transfection measured by Colony formation assay. **l**, PC3 cell apoptosis following varied transfection measured by flow cytometry. **m**, PC3 cell invasion following varied transfection measured by Transwell assay. Scale bar = 50 μm. **n**, PC3 cell migration following varied transfection measured by Scratch test. * *p* < 0.05 vs. adjacent normal tissues, RWPE-1 cells, or cells transfected with oe-NC or oe-NC + mimic NC. # *p* < 0.05 vs. cells transfected with oe-KDM5A + mimic NC. ^ns^ *p >* 0.05 vs. cells transfected with oe-NC + miR-495 mimic or oe-KDM5A + mimic NC. Data in panel A were analyzed by paired *t*-test, in panel D-G by unpaired *t*-test and those in panel H-J were analyzed by one-way ANOVA with Tukey’s post hoc test

We proceeded to examine the specific mechanism by which KDM5A regulates biological and physiological functions of PCa cells. ChIP-seq analysis showed that KDM5A could bind to the promoter of miR-495, suggesting that KDM5A might play a role in the development of PCa by targeting miR-495. miR-495 was detected to be decreased in PCa tissues using RT-qPCR (*p* < 0.05; Fig. 3a), and was negatively correlated with the mRNA expression level of KDM5A (Fig. 3b). In addition, miR-495 expression level was lower in LNCaP, C42, DU145 and PC3 cells than in RWPE-1 cells (*p* < 0.05; Fig. 3c). Dual luciferase reporter assay showed that the luciferase activity of miR-495 promoter-WT was decreased following oe-KDM5A transfection (*p* < 0.05) while no statistical changes were observed in luciferase activity of miR-495 promoter-MUT (*p* > 0.05; Fig. 3d), indicating that KDM5A could bind to the miR-495 promoter. Moreover, miR-495 expression was reduced in cells transfected with oe-KDM5A (*p* < 0.05; Fig. 3e). ChIP assay also confirmed the binding of KDM5A to miR-495 promoter region since the binding of miR-495 to KDM5A protein was increased following the overexpression of KDM5A (*p* < 0.05; Fig. 3f). Western blots further demonstrated that H3K4me3 expression in the antibody-transcription factor complex pulled down by KDM5A antibody was decreased (*p* < 0.05; Fig. 3g). These data indicate that KDM5A, as a H3K4me3 demethylase, can bind to the promoter region of miR-495 to suppress its transcription, and then decrease its expression in human PC3 cells.

In order to verify whether KDM5A functions in the occurrence and development of PCa by regulating miR-495, we performed a series of in vitro experiments. The results of RT-qPCR, Western blot analysis (Fig. 3h-j), colony formation assay (Fig. 3k), flow cytometry (Fig. 3l), Transwell assay (Fig. 3m) and Scratch test (Fig. 3n) and showed no significant changes in the mRNA and protein expression of KDM5A, yet increased miR-495 expression and cell apoptosis, reduced cell proliferation, invasion and migration in PC3 cells overexpressing miR-495. The transfection with oe-KDM5A in cells, however, resulted in an increase in the mRNA and protein expression of KDM5A as well as cell proliferation, migration and invasion, in addition to a reduction in miR-495 expression and cell apoptosis (*p* < 0.05). Co-transfection with oe-KDM5A and miR-495 mimic found no statistical alterations in KDM5A expression, elevated miR-495 expression, decreased cell proliferation, migration and invasion yet enhanced cell apoptosis (*p* < 0.05). Taken together, KDM5A could promote the proliferation, invasion and migration of PCa cells and reduces cell apoptosis by downregulating the expression of miR-495.

### YTHDF2 is a target gene of miR-495

**Fig. 4:**
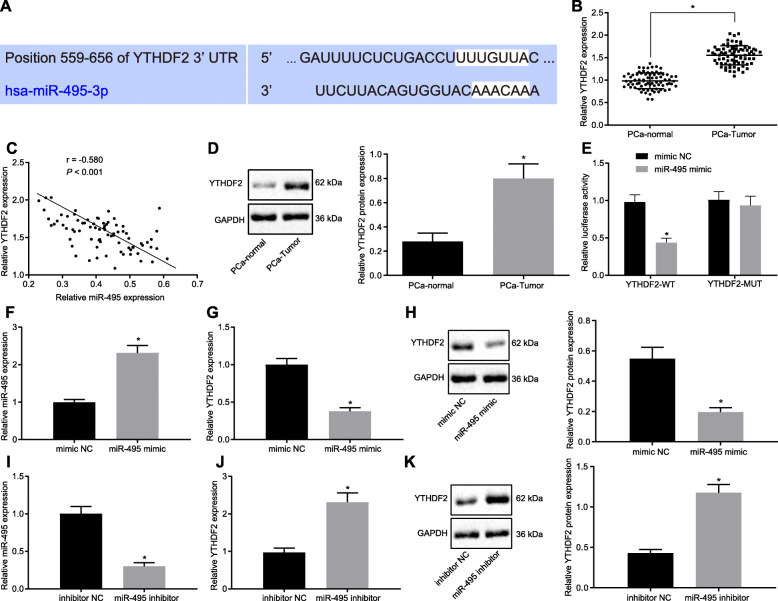
miR-495 targets YTHDF2 and suppresses its expression. **a**, Predicted binding sites between miR-495 and YTHDF2 via bioinformatics analysis. **b**, The mRNA expression of YTHDF2 in PCa and adjacent normal tissues detected by RT-qPCR. **c**, Correlation between miR-495 and YTHDF2 in PCa tissues and adjacent normal tissues of the patients. **d**, Western blot analysis of YTHDF2 protein in cancer and adjacent normal tissues of the patients. **e**, The binding of miR-495 to the mRNA of YTHDF2 confirmed by dual luciferase reporter assay. **f** and **i**, miR-495 expression in miR-495 mimic- or miR-495 inhibitor-transfected cells detected by RT-qPCR. G and **j**, YTHDF2 mRNA expression in miR-495 mimic- or miR-495 inhibitor-transfected cells detected by RT-qPCR. **h** and **k**, Western blot analysis and quantification of YTHDF2 protein in miR-495 mimic- or miR-495 inhibitor-transfected cells. * *p* < 0.05 vs. adjacent normal tissues, or cells transfected with mimic NC or inhibitor NC. Data in panel B and D were analyzed using paired *t*-test, and those in panel E-K were analyzed using unpaired *t*-test

Having identified the role of KDM5A in promoting PCa cell proliferation, invasion and migration through inhibition of miR-495, we moved our attention on the underlying mechanism of miR-495 in PCa. Bioinformatics analysis predicted the binding sites between miR-495 and YTHDF2 (Fig. 4a). RT-qPCR detected that the mRNA expression of YTHDF2 was increased (*p* < 0.05; Fig. 4b), while miR-495 expression was reduced in patient cancer tissues (*p* < 0.05; Fig. 4c). Moreover, YTHDF2 protein expression was also increased in patient PCa tissues (*p* < 0.05; Fig. 4d). Based on the above results, we speculated that miR-495 might be involved in the pathogenesis of PCa by targeting YTHDF2. The results of dual luciferase reporter showed a decline in the luciferase activity of YTHDF2-MUT (*p* < 0.05) while YTHDF2-MUT luciferase activity was unaffected following miR-495 mimic transfection (*p* > 0.05; Fig. 4e), indicating that miR-495 could combine with YTHDF2 mRNA. RT-qPCR and Western blots revealed upregulated miR-495 expression (*p* < 0.05; Fig. 4f) and downregulated YTHDF2 mRNA and protein expression (*p* < 0.05; Fig. 4g, h) in miR-495 mimic-transfected cells, which was reversed by transfection with miR-495 inhibitor (*p* < 0.05; Fig. 4i-k). These results indicate that miR-495 can target YTHDF2 and inhibit its expression in PCa cells.

### Overexpression YTHDF2 could reverse the inhibitory effect of miR-495 on PCa cell proliferation, invasion and migration

**Fig. 5:**
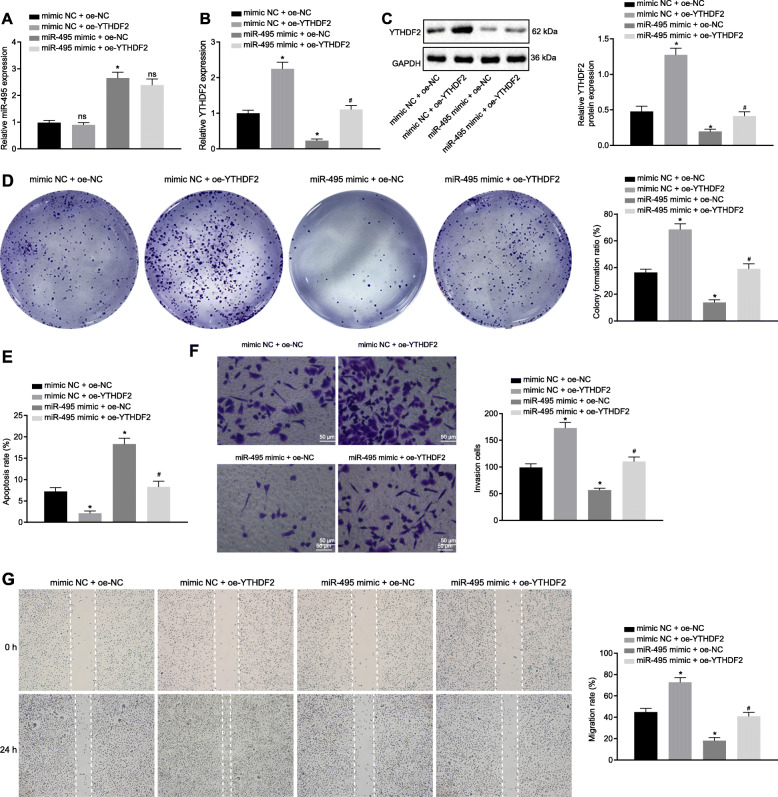
Upregulated YTHDF2 promotes the inhibited proliferation, invasion and migration while diminishing the augmented cell apoptosis evoked by miR-495 in PCa cells. **a**, miR-495 expression in cells following varied transfection detected by RT-qPCR. **b**, The mRNA expression of YTHDF2 in cells following varied transfection detected by RT-qPCR. **c**, Western blots of YTHDF2 protein in cells following varied transfection. **d**, PC3 cell proliferation following varied transfection measured by Colony formation assay. **e**, PC3 cell apoptosis following varied transfection measured by flow cytometry. **f**, PC3 cell invasion following varied transfection measured by Transwell assay. Scale bar = 50 μm. **g**, PC3 cell migration following varied transfection measured by Scratch test. * *p* < 0.05 vs. cells transfected with mimic NC + oe-NC. # *p* < 0.05 vs. cells transfected with miR-495 mimic + oe-NC. ^ns^ *p >* 0.05 vs. cells transfected with mimic NC + oe-NC or miR-495 mimic + oe-NC. Data in panel A-G were analyzed by one-way ANOVA with Tukey’s post hoc test

We then investigated whether miR-495 can inhibit the proliferation, invasion and migration of PCa cells by targeting YTHDF2. Overexpression of YTHDF2 alone led to no statistical changes in the expression of miR-495 (*p* > 0.05; Fig. 5a), but increased mRNA and protein expression of YTHDF2 (*p* < 0.05; Fig. 5b, c), enhanced cell proliferation (*p* < 0.05; Fig. 5d), reduced cell apoptosis (*p* < 0.05; Fig. 5e), and facilitated cell migration and invasion (*p* < 0.05; Fig. 5f, g) and. By contrast, miR-495 mimic-transfected cells exhibited miR-495 overexpression, decreased mRNA and protein expression of YTHDF2, diminished cell proliferation, migration and invasion yet augmented apoptosis. In addition, transfection with both miR-495 mimic and oe-YTHDF2 presented with similar results as those following oe-YTHDF2 transfection alone. In summary, overexpression of YTHDF2 can undermine the inhibitory effect of miR-495 on the proliferation, invasion and migration of PCa cells, and the promoting effect in cell apoptosis.

### YTHDF2 recognizes the m6A modification of MOB3B mRNA, induces the mRNA degradation and downregulates MOB3B expression

**Fig. 6:**
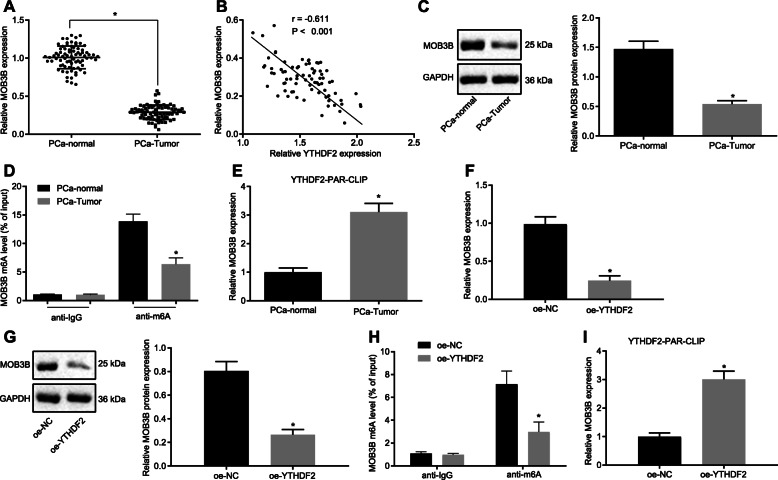
YTHDF2 recognizes the m6A modification of MOB3B mRNA, evokes mRNA degradation and suppresses MOB3B expression. **a**, MOB3B mRNA expression in cancer and adjacent normal tissues of 75 patients detected by RT-qPCR. **b**, Correlation between MOB3B and YTHDF2 in cancer and adjacent normal tissues of 75 patients. **c**, Western blot analysis of MOB3B protein in cancer and adjacent normal tissues of 75 patients. **d**, m6A modification level of MOB3B in cancer and adjacent normal tissues assessed using Me-RIP. **e**, The binding of YTHDF2 to the MOB3B mRNA in cancer and adjacent normal tissues confirmed by PAR-CLIP assay. **f**, MOB3B mRNA expression in oe-YTHDF2-transfected PC3 cells detected by RT-qPCR. **g**, Western blot analysis of MOB3B protein in oe-YTHDF2-transfected PC3 cells. **h**, m6A modification in MOB3B in oe-YTHDF2-transfected PC3 cells assessed using Me-RIP. **i**, The binding of YTHDF2 to the MOB3B mRNA in oe-YTHDF2-transfected PC3 cells confirmed by PAR-CLIP assay. * *p* < 0.05 vs. adjacent normal tissues or cells transfected with oe-NC. Data in panel A and C-E were analyzed by paired *t*-test and those in panel F-I were analyzed by unpaired *t*-test

We further elaborate the mechanism of YTHDF2 in PCa. RT-qPCR showed that MOB3B mRNA expression level was decreased in PCa tissues (*p* < 0.05; Fig. 6a), while YTHDF2 mRNA expression was elevated (*p* < 0.05; Fig. 6b). Western blots also detected a decline in the protein expression of MOB3B in PCa tissues (*p* < 0.05; Fig. 6c). In addition, the m6A modification level of MOB3B was reduced in PCa tissues (*p* < 0.05; Fig. 6d). The PAR-CLIP revealed that mRNA expression of MOB3B binding to YTHDF2 was increased in PCa tissues (*p* < 0.05; Fig. 6e). As shown in Fig. 6f, g, cells transfected with oe-YTHDF2 exhibited downregulated mRNA and protein expression of MOB3B (*p* < 0.05), decreased m6A modification level of MOB3B (*p* < 0.05; Fig. 6h), and significantly more binding of YTHDF2 to the MOB3B mRNA (*p* < 0.05; Fig. 6i). These results indicate that YTHDF2 can recognize the m6A modification of MOB3B mRNA, promote the degradation of mRNA and inhibit MOB3B expression.

### KDM5A inhibits MOB3B, drives PCa cell proliferation, migration and invasion, and attenuates cell apoptosis via the miR-495/YTHDF2 axis

**Fig. 7:**
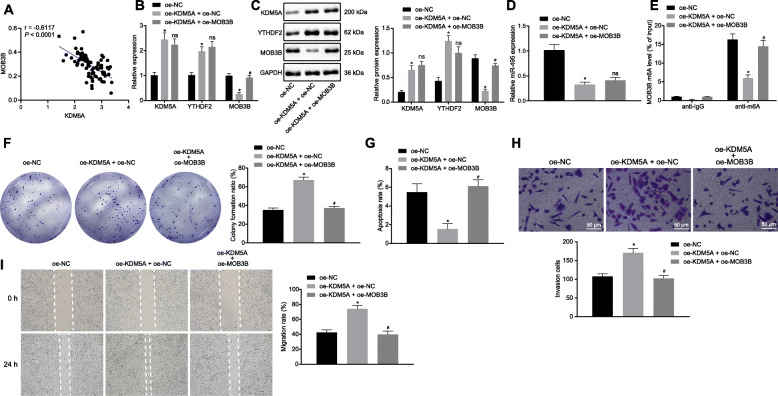
KDM5A represses MOB3B and then elicits proliferation, migration and invasion of PCa cells while reducing cell apoptosis via the miR-495/YTHDF2 axis. **a**, Correlation analysis of KDM5A and MOB3B in PCa tissues. **b**, The mRNA expression of KDM5A, YTHDF2 and MOB3B in PC3 cells with varied transfection detected by RT-qPCR. **c**, Western blots of KDM5A, YTHDF2 and MOB3B proteins in cells with varied transfection. **d**, miR-495 expression in cells following varied transfection detected by RT-qPCR. **e**, The m6A modification level of MOB3B in cells following varied transfection assessed using Me-RIP. **f**, PC3 cell proliferation following varied transfection measured by Colony formation assay. **g**, PC3 cell apoptosis following varied transfection measured by flow cytometry. **h**, PC3 cell invasion following varied transfection measured by Transwell assay. Scale bar = 50 μm. **i**, PC3 cell migration following varied transfection measured by Scratch test (Scale bar = 200 μm). * *p* < 0.05 vs. cells transfected with oe-NC. # *p* < 0.05 or ^ns^ *p* > 0.05 vs. cells transfected with oe-KDM5A + oe-NC. Data in panel **a-h** were analyzed by one-way ANOVA with Tukey’s post hoc test

We extended our mechanistic findings to determine whether KDM5A regulates the expression of MOB3B via the miR-495/YTHDF2 axis to promote the proliferation, migration and invasion of the PCa cells. We analyzed the correlation between KDM5A and MOB3B expression in PCa tissues from 78 PCa patients, and the results showed that there was a significant negative correlation between KDM5A and MOB3B (*p* < 0.05) (Fig. 7a). In PC3 cells transfected with oe-KDM5A, the mRNA and protein levels of KDM5A and YTHDF2 were increased (*p* < 0.05; Fig. 7b, c), while miR-495 expression and MOB3B mRNA and protein expression levels were decreased (*p* < 0.05; Fig. 7d). We observed the m6A modification level of MOB3B mRNA was diminished (*p* < 0.05; Fig. 7e), cell proliferation was enhanced (*p* < 0.05; Fig. 7f), cell apoptosis was reduced (*p* < 0.05; Fig. 7g), cell invasion and migration was facilitated (*p* < 0.05; Fig. 7h, i) in response to oe-KDM5A transfection. Co-transfection with oe-KDM5A and oe-MOB3B resulted in no alterations in expression of KDM5A, miR-495 and YTHDF2 (*p* > 0.05), but increased MOB3B mRNA and protein expression as well as m6A modification level of MOB3B mRNA, enhanced cell apoptosis, while attenuating cell proliferation, invasion and migration (*p* < 0.05). The above results illustrate that KDM5A can upregulate YTHDF2 expression by inhibiting miR-495, thus downregulating MOB3B expression, whereby subsequently stimulating the proliferation, migration and invasion of the PCa cells and reducing cell apoptosis.

### KDM5A suppresses MOB3B and promotes tumorigenicity in nude mice via the miR-495/YTHDF2 axis

**Fig. 8:**
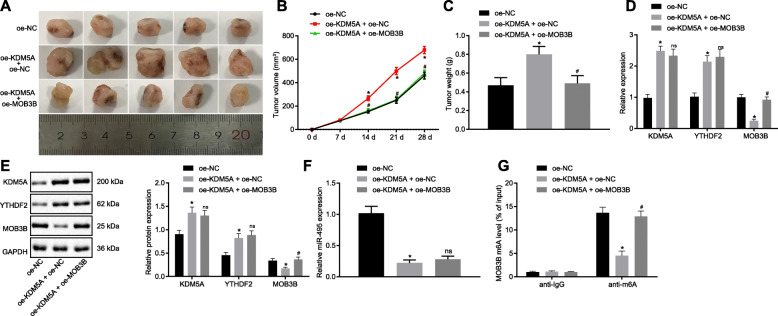
KDM5A promotes tumorigenicity by downregulating MOB3B expression in nude mice via the miR-495/YTHDF2 axis. **a-c**, Xenograft tumors and quantitative analysis of tumor size and volume after oe-KDM5A or oe-KDM5A + oe-MOB3B treatment. Scale bar = 10 mm in panel A. **d**, mRNA expression of KDM5A, YTHDF2 and MOB3B in mice following oe-KDM5A or oe-KDM5A + oe-MOB3B treatment detected by RT-qPCR. **e**, Western blots of KDM5A, YTHDF2 and MOB3B proteins in mice following oe-KDM5A or oe-KDM5A + oe-MOB3B treatment. **f**, miR-495 expression in mice following oe-KDM5A or oe-KDM5A + oe-MOB3B treatment detected by RT-qPCR. **g**, m6A modification level of MOB3B in mice following oe-KDM5A or oe-KDM5A + oe-MOB3B treatment assessed using Me-RIP. * *p* < 0.05 vs. cells transfected with oe-NC. # *p* < 0.05 or ^ns^ *p* > 0.05 vs. cells transfected with oe-KDM5A + oe-NC. Data in panel A and C-G were analyzed by one-way ANOVA with Tukey’s post hoc test, and those in panel B were analyzed by repeated measures ANOVA with Bonferroni’s post hoc test

We carried out xenograft tumor experiments on the nude mice in order to verify the effect of KDM5A on PCa initiation and progression via the miR-495/YTHDF2/MOB3B axis in vivo. The mice treated with oe-KDM5A presented with increased tumor size and weight (*p* < 0.05; Fig. 8a-c), upregulated mRNA and protein expression of KDM5A and YTHDF2 (*p* < 0.05; Fig. 8d, e), and decreased miR-495 expression (*p* < 0.05; Fig. 8f) and MOB3B mRNA and protein expression as well as diminished m6A modification level of MOB3B mRNA (*p* < 0.05; Fig. 8g). Treatment with oe-KDM5A and oe-MOB3B, however, resulted in decreased tumor size and weight (*p* < 0.05), increased MOB3B mRNA and protein expression and m6A modification level of MOB3B mRNA (*p* < 0.05) while no statistical changes were observed in miR-495 expression, mRNA and protein expression of KDM5A and YTHDF2 (*p* > 0.05). These data indicate that KDM5A represses MOB3B and facilitates tumorigenicity in nude mice via the miR-495/YTHDF2 axis.

## Discussion

Histone lysine demethylases have emerged as novel prognostic factors and therapeutic targets for prophylaxis and management of advanced PCa [13]. In the present study, we investigated the cancer-promoting mechanism of KDM5A underlying the pathogenesis of PCa. The obtained in vitro and in vivo experimental results demonstrated that KDM5A elicited PCa progression via downregulation of miR-495 expression and YTHDF2-mediated MOB3B inhibition.

KDM5A gene is significantly amplified and overexpressed in various human cancers, such as ovarian cancer, small cell lung cancer and breast cancer [4, 14, 15]. In the present study, KDM5A was found to be expressed at high levels in human PCa tissues and cell lines. Similarly, Vieira group found that the upregulated KDM5A expression in PCa tissues compared with normal prostate tissues, but its downstream mechanism has not been fully elucidated [8]. Our study showed that upregulated KDM5A could promote proliferation, migration and invasion of PCa cells while reducing cell apoptosis via the inhibition of miR-495. It has been reported that KDM5A downregulates miR-21 expression in leukemia cells by directly binding to the promoter sequence of miR-21 and demethylating trimethylated H3K4 at the miR-21 locus [16]. Consistent with our findings, ChIP-seq data revealed that KDM5A could bind to the promoter of miR-495 and consequently repress its expression.

Accumulating evidence has identified miR-495 to be a tumor suppressor miRNA in PCa. miR-495 is decreased in PCa cell lines compared with normal prostatic epithelial cells [17]. Moreover, amplified miR-495 has been found to inhibit PCa cell proliferation, migration, and invasion in vitro as well as significantly retarding the growth of tumors in vivo by repressing its targets Akt and mechanistic target of rapamycin (mTOR) [9]. miRNAs can modulate gene expression posttranscriptionally by interacting with the 3′-untranslated region (UTR) of specific target mRNAs [18]. In the present study, we confirmed that YTHDF2 is a target gene of miR-495 and could be negatively regulated by it. Chen et al. found that mRNA and protein expression of YTHDF2 is upregulated in PCa tissues compared with adjacent normal tissues, which can promote cell proliferation of prostate tissues [19]. Recent evidence demonstrates that miRNAs play an important role in regulation of cancer cell growth, invasion and metastasis through suppression of the expression of their target genes [20]. Those findings thus supported our conclusion that upregulated YTHDF2 abolished the inhibitory effects of miR-495 on PCa cell functions of proliferation, invasion and migration and the inducing effects on cell apoptosis.

The m6A reader protein YTHDF2 has been previous reported to promote mRNA degradation by recognizing m6A and recruiting the mRNA decay machinery [11]. More specifically, following the binding of YTHDF2 to m6A sites, the recognized mRNAs are degraded and m6A levels are decreased accordingly [10]. The findings from our present study revealed that YTHDF2 might recognize m6A modification of MOB3B mRNA and then enhance the mRNA degradation, ultimately disrupting the expression of MOB3B. In accordance to our results, the mRNA expression of MOB3B is significantly lower in PCa tissues than in healthy control tissues, which is further closely associated with aggressive pathophysiological features in patients with PCa [21]. By directly binding to the promoter region of MOB1, KDM2B has been demonstrated to suppress the promoter activity of MOB1 and transcriptionally inhibit MOB1 expression, thus augmenting the proliferation, migration and invasion of pancreatic ductal adenocarcinoma cells [22], which is partially in consistent with our results. Furthermore, in the response of both PC3 cell line and nude mice to KDM5A overexpression, the mRNA and protein levels of YTHDF2 was found to be significantly downregulated. We concluded that KDM5A stimulated PCa initiation by downregulating MOB3B expression via regulation of the miR-495/YTHDF2 axis both in vivo and in vitro. However, limitation exists. it will be good to compare PC3 cells with other PCa cells, such as LNCaP and DU145 cells in future studies, so as to validate the effects in PCa cells with different metastatic potential and different survival and proliferation characteristics. Moreover, the in vitro findings could also be verified with future confirmatory experiments in normal prostate epithelial cells.

## Conclusion

In summary, our study demonstrates that the overexpression of KDM5A has a tumor-supporting effect on PCa, as it suppresses the YTHDF2-dependent MOB3B expression by binding to miR-495 (Supplementary Fig. 1). These findings may assist with potential future therapeutic strategies of PCa prevention and treatment. Nevertheless, more in-depth investigations as well as combined research efforts are still required in the near future to validate its applicable values into clinical practice.

## Supplementary information

**Additional file 1 Supplementary Figure 1** Schematic diagram showing that KDM5A downregulates MOB3B via the miR-495/YTHDF2 axis, which enhances the progression of PCa.

## Data Availability

The datasets generated and/or analyzed during the current study are available from the corresponding author on reasonable request.

## Abbreviations

KDM5: Lysine-specific demethylase 5
KDM5A: Lysine-specific demethylase 5A
MPC-1: Mitochondrial pyruvate carrier 1
miRs or miRNAs: MicroRNAs
YTHDF2: YTH domain family 2
MOB3B: Mps one binder kinase activator 3B
OS: Total survival
DFS: Progression-free survival
GAPDH: Glyceraldehyde-3-phosphate dehydrogenase
sh: Short hairpin
oe: Overexpression
FITC: Fluorescein isothiocyanate
PI: Propidium iodide
WT: Promoter-wild-type
MUT: miR-495 promoter-mutant
RLU: Relative luciferase unit
ChIP: Chromatin immunoprecipitation
Me-RIP: Methylated RNA binding protein immunoprecipitation
PAR-CLIP: Photo-activatable ribonucleoside-enhanced crosslinking and immunoprecipitation
ANOVA: Analysis of variance
